# Dichlorido(1,10-phenanthroline-κ^2^ *N*,*N*′)palladium(II)

**DOI:** 10.1107/S1600536809052313

**Published:** 2009-12-12

**Authors:** Kwang Ha

**Affiliations:** a School of Applied Chemical Engineering, The Research Institute of Catalysis, Chonnam National University, Gwangju 500-757, Republic of Korea

## Abstract

In the title complex, [PdCl_2_(C_12_H_8_N_2_)], the Pd^2+^ ion is four-coordinated in a slightly distorted square-planar environment by two N atoms of the chelating 1,10-phenanthroline ligand and two chloride ions. The nearly planar mol­ecules, with a maximum deviation of 0.120 (3) Å from the least-squares plane, are stacked in columns along the *c* axis with a Pd⋯Pd distance of 4.8340 (9) Å. In the column, π–π inter­actions between adjacent six-membered rings are present, the shortest centroid–centroid distance being 3.680 (4) Å. A weak C—H⋯Cl inter­action is observed between the columns.

## Related literature

For the syntheses of [Pd*X*_2_(phen)] (phen = 1,10-phenanthroline; *X* = Cl, Br, I or SCN), see: Cheng *et al.* (1977 ▶). For the crystal structure of yellow [PtCl_2_(phen)] which is isotypic to the title complex, see: Grzesiak & Matzger (2007 ▶). For the crystal structures of related Pd–bipy complexes, [Pd*X*_2_(bipy)] (bipy = 2,2′-bipyridine; *X* = Cl, Br or I), see: Maekawa *et al.* (1991 ▶); Smeets *et al.* (1997 ▶); Ha (2009 ▶).
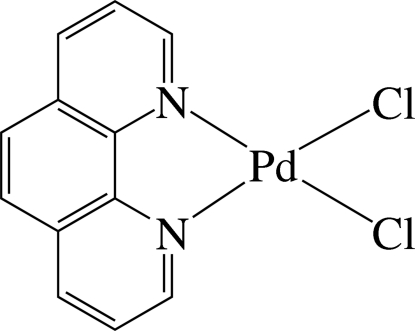


## Experimental

### 

#### Crystal data

[PdCl_2_(C_12_H_8_N_2_)]
*M* *_r_* = 357.50
Monoclinic, 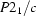

*a* = 9.6170 (8) Å
*b* = 17.1402 (14) Å
*c* = 7.2529 (6) Å
β = 109.314 (2)°
*V* = 1128.26 (16) Å^3^
*Z* = 4
Mo *K*α radiation
μ = 2.09 mm^−1^
*T* = 200 K
0.36 × 0.06 × 0.04 mm

#### Data collection

Bruker SMART 1000 CCD diffractometer
Absorption correction: multi-scan (*SADABS*; Bruker, 2000 ▶) *T*_min_ = 0.769, *T*_max_ = 0.920
8118 measured reflections
2767 independent reflections
1852 reflections with *I* > 2σ(*I*)
*R*_int_ = 0.060

#### Refinement

*R*[*F* ^2^ > 2σ(*F* ^2^)] = 0.056
*wR*(*F* ^2^) = 0.169
*S* = 1.10
2767 reflections
154 parameters
H-atom parameters constrained
Δρ_max_ = 2.90 e Å^−3^
Δρ_min_ = −1.29 e Å^−3^

### 

Data collection: *SMART* (Bruker, 2000 ▶); cell refinement: *SAINT* (Bruker, 2000 ▶); data reduction: *SAINT*; program(s) used to solve structure: *SHELXS97* (Sheldrick, 2008 ▶); program(s) used to refine structure: *SHELXL97* (Sheldrick, 2008 ▶); molecular graphics: *ORTEP-3* (Farrugia, 1997 ▶) and *PLATON* (Spek, 2009 ▶); software used to prepare material for publication: *SHELXL97*.

## Supplementary Material

Crystal structure: contains datablocks global, I. DOI: 10.1107/S1600536809052313/is2500sup1.cif

Structure factors: contains datablocks I. DOI: 10.1107/S1600536809052313/is2500Isup2.hkl

Additional supplementary materials:  crystallographic information; 3D view; checkCIF report

## Figures and Tables

**Table:** 

Pd1—N2	2.035 (6)
Pd1—N1	2.036 (6)
Pd1—Cl2	2.283 (2)
Pd1—Cl1	2.2914 (19)

**Table:** 

N2—Pd1—N1	81.5 (2)

**Table 2:** Hydrogen-bond geometry (Å, °)

*D*—H⋯*A*	*D*—H	H⋯*A*	*D*⋯*A*	*D*—H⋯*A*
C2—H2⋯Cl1^i^	0.95	2.80	3.733 (11)	169

Symmetry code: (i) 
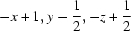
.

**Fig. 1.**
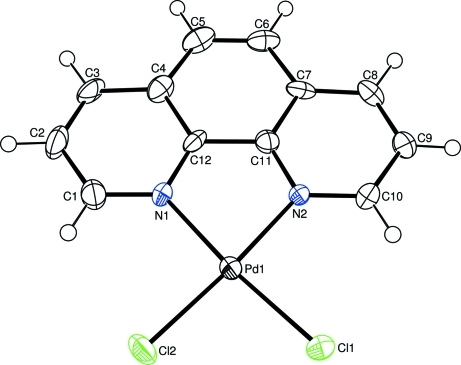
The structure of the title complex, with displacement ellipsoids drawn at the 50% probability level for non-H atoms.

**Fig. 2.**
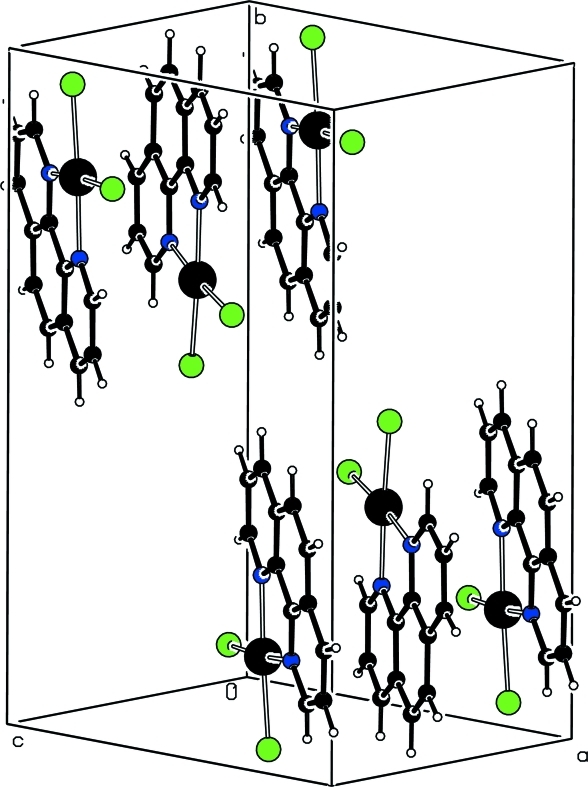
Crystal packing diagram of the title complex.

## supplementary crystallographic information

### Comment

The title complex, [PdCl_2_(phen)] (where phen is 1,10-phenanthroline, C_12_H_8_N_2_), is isomorphous with the yellow form of [PtCl_2_(phen)], whereas the orange form of [PtCl_2_(phen)] crystallized in the orthorhombic space group *Pca*2_1_ (Grzesiak & Matzger, 2007).

In the title complex, the Pd^2+^ ion is four-coordinated in a slightly distorted square-planar environment by two N atoms of the chelating 1,10-phenanthroline ligand and two chloride ions (Fig. 1). The main contribution to the distortion is the tight N1—Pd1—N2 chelate angle [81.5 (2)°], which results in non-linear *trans* arrangement [<N1—Pd1—Cl1 = 174.64 (17)° and <N2—Pd1—Cl2 = 175.11 (17)°]. The Pd1—N and Pd1—Cl bond lengths are almost equal, respectively [Pd1—N: 2.036 (6) and 2.035 (6) Å; Pd1—Cl 2.2914 (19) and 2.283 (2) Å]. The complex displays numerous intermolecular π-π interactions between adjacent six-membered rings, with a shortest centroid-centroid distance of 3.680 (4) Å and the dihedral angle between the ring planes is 5.6 (4)°. The nearly planar [PdCl_2_(phen)] molecules with a largest deviation of 0.120 (3) Å from the least-squares plane stack in columns along the *c* axis with a Pd···Pd distance of 4.8340 (9) Å (Fig. 2).

### Experimental

To a solution of Na_2_PdCl_4_ (0.5095 g, 1.732 mmol) in H_2_O (40 ml) was added 1,10-phenanthroline (0.3122 g, 1.732 mmol) and refluxed for 3 h. The precipitate obtained was separated by filtration, washed with water and acetone, and dried at 70 °C, to give a pale yellow powder (0.5259 g). Crystals suitable for X-ray analysis were obtained by slow evaporation from a dimethyl sulfoxide solution at 80 °C.

### Refinement

H atoms were positioned geometrically and allowed to ride on their respective parent atoms [C—H = 0.95 Å and *U*_iso_(H) = 1.2*U*_eq_(C)]. The highest peak and the deepest hole in the difference Fourier map are located 1.14 and 0.85 Å, respectively, from atom Pd1.

### Crystal data

**Table:**

[PdCl_2_(C_12_H_8_N_2_)]	*F*(000) = 696
*M**_r_* = 357.50	*D*_x_ = 2.105 Mg m^−^^3^
Monoclinic, *P*2_1_/*c*	Mo *K*α radiation, λ = 0.71073 Å
Hall symbol: -P 2ybc	Cell parameters from 2653 reflections
*a* = 9.6170 (8) Å	θ = 2.4–28.2°
*b* = 17.1402 (14) Å	µ = 2.09 mm^−^^1^
*c* = 7.2529 (6) Å	*T* = 200 K
β = 109.314 (2)°	Needle, yellow
*V* = 1128.26 (16) Å^3^	0.36 × 0.06 × 0.04 mm
*Z* = 4	

### Data collection

**Table:**

Bruker SMART 1000 CCD diffractometer	2767 independent reflections
Radiation source: fine-focus sealed tube	1852 reflections with *I* > 2σ(*I*)
graphite	*R*_int_ = 0.060
φ and ω scans	θ_max_ = 28.3°, θ_min_ = 2.2°
Absorption correction: multi-scan (*SADABS*; Bruker, 2000)	*h* = −12→12
*T*_min_ = 0.769, *T*_max_ = 0.920	*k* = −22→19
8118 measured reflections	*l* = −9→9

### Refinement

**Table:**

Refinement on *F*^2^	Primary atom site location: structure-invariant direct methods
Least-squares matrix: full	Secondary atom site location: difference Fourier map
*R*[*F*^2^ > 2σ(*F*^2^)] = 0.056	Hydrogen site location: inferred from neighbouring sites
*wR*(*F*^2^) = 0.169	H-atom parameters constrained
*S* = 1.10	*w* = 1/[σ^2^(*F*_o_^2^) + (0.084*P*)^2^] where *P* = (*F*_o_^2^ + 2*F*_c_^2^)/3
2767 reflections	(Δ/σ)_max_ < 0.001
154 parameters	Δρ_max_ = 2.90 e Å^−^^3^
0 restraints	Δρ_min_ = −1.29 e Å^−^^3^

### Special details

**Table:**

Geometry. All e.s.d.'s (except the e.s.d. in the dihedral angle between two l.s. planes) are estimated using the full covariance matrix. The cell e.s.d.'s are taken into account individually in the estimation of e.s.d.'s in distances, angles and torsion angles; correlations between e.s.d.'s in cell parameters are only used when they are defined by crystal symmetry. An approximate (isotropic) treatment of cell e.s.d.'s is used for estimating e.s.d.'s involving l.s. planes.
Refinement. Refinement of *F*^2^ against ALL reflections. The weighted *R*-factor *wR* and goodness of fit *S* are based on *F*^2^, conventional *R*-factors *R* are based on *F*, with *F* set to zero for negative *F*^2^. The threshold expression of *F*^2^ > σ(*F*^2^) is used only for calculating *R*-factors(gt) *etc*. and is not relevant to the choice of reflections for refinement. *R*-factors based on *F*^2^ are statistically about twice as large as those based on *F*, and *R*- factors based on ALL data will be even larger.

### Fractional atomic coordinates and isotropic or equivalent isotropic displacement parameters (Å^2^)

**Table:**

	*x*	*y*	*z*	*U*_iso_*/*U*_eq_	
Pd1	0.70739 (6)	0.34324 (3)	0.39119 (8)	0.0214 (2)	
Cl1	0.7453 (2)	0.47533 (11)	0.4189 (3)	0.0313 (5)	
Cl2	0.4658 (2)	0.36052 (12)	0.2078 (3)	0.0366 (5)	
N1	0.6941 (6)	0.2247 (3)	0.3805 (9)	0.0235 (13)	
N2	0.9182 (6)	0.3184 (3)	0.5594 (9)	0.0211 (13)	
C1	0.5819 (9)	0.1794 (5)	0.2838 (12)	0.0319 (18)	
H1	0.4931	0.2030	0.2036	0.038*	
C2	0.5898 (10)	0.0983 (5)	0.2958 (13)	0.039 (2)	
H2	0.5073	0.0676	0.2244	0.046*	
C3	0.7163 (9)	0.0629 (4)	0.4102 (12)	0.033 (2)	
H3	0.7214	0.0076	0.4202	0.040*	
C4	0.8382 (9)	0.1085 (4)	0.5127 (11)	0.0286 (17)	
C5	0.9777 (10)	0.0785 (4)	0.6369 (12)	0.0330 (19)	
H5	0.9904	0.0238	0.6561	0.040*	
C6	1.0895 (9)	0.1263 (5)	0.7254 (12)	0.0318 (18)	
H6	1.1805	0.1045	0.8044	0.038*	
C7	1.0771 (8)	0.2089 (4)	0.7057 (11)	0.0263 (16)	
C8	1.1907 (8)	0.2626 (5)	0.7913 (12)	0.0301 (17)	
H8	1.2850	0.2445	0.8702	0.036*	
C9	1.1660 (8)	0.3405 (4)	0.7613 (12)	0.0287 (17)	
H9	1.2423	0.3768	0.8210	0.034*	
C10	1.0283 (8)	0.3669 (4)	0.6426 (11)	0.0269 (16)	
H10	1.0131	0.4213	0.6207	0.032*	
C11	0.9420 (8)	0.2407 (4)	0.5896 (10)	0.0234 (15)	
C12	0.8212 (8)	0.1902 (4)	0.4927 (11)	0.0224 (15)	

### Atomic displacement parameters (Å^2^)

**Table:**

	*U*^11^	*U*^22^	*U*^33^	*U*^12^	*U*^13^	*U*^23^
Pd1	0.0171 (3)	0.0198 (3)	0.0258 (3)	−0.0001 (2)	0.0050 (2)	0.0001 (2)
Cl1	0.0285 (10)	0.0198 (9)	0.0422 (12)	0.0034 (7)	0.0070 (9)	0.0016 (7)
Cl2	0.0178 (9)	0.0379 (11)	0.0457 (13)	0.0017 (8)	−0.0007 (8)	0.0059 (9)
N1	0.022 (3)	0.016 (3)	0.031 (3)	−0.003 (2)	0.006 (3)	−0.004 (2)
N2	0.018 (3)	0.014 (3)	0.030 (3)	0.000 (2)	0.006 (3)	0.000 (2)
C1	0.026 (4)	0.037 (4)	0.031 (4)	−0.005 (3)	0.007 (4)	−0.005 (3)
C2	0.040 (5)	0.029 (5)	0.048 (5)	−0.017 (4)	0.016 (4)	−0.015 (4)
C3	0.043 (5)	0.011 (4)	0.053 (6)	−0.005 (3)	0.025 (5)	−0.004 (3)
C4	0.038 (5)	0.022 (4)	0.030 (4)	−0.005 (3)	0.018 (4)	0.000 (3)
C5	0.054 (6)	0.021 (4)	0.029 (4)	0.005 (4)	0.021 (4)	0.006 (3)
C6	0.039 (5)	0.026 (4)	0.035 (5)	0.013 (4)	0.019 (4)	0.008 (3)
C7	0.023 (4)	0.026 (4)	0.034 (4)	0.011 (3)	0.015 (3)	0.007 (3)
C8	0.016 (4)	0.039 (5)	0.033 (5)	0.003 (3)	0.006 (3)	0.005 (3)
C9	0.020 (4)	0.032 (4)	0.029 (4)	−0.001 (3)	0.001 (3)	−0.003 (3)
C10	0.029 (4)	0.021 (4)	0.031 (4)	−0.003 (3)	0.009 (3)	−0.004 (3)
C11	0.022 (4)	0.026 (4)	0.023 (4)	0.000 (3)	0.009 (3)	−0.003 (3)
C12	0.032 (4)	0.013 (3)	0.027 (4)	−0.003 (3)	0.016 (3)	−0.005 (3)

### Geometric parameters (Å, °)

**Table:**

Pd1—N2	2.035 (6)	C4—C12	1.412 (10)
Pd1—N1	2.036 (6)	C4—C5	1.441 (12)
Pd1—Cl2	2.283 (2)	C5—C6	1.335 (11)
Pd1—Cl1	2.2914 (19)	C5—H5	0.9500
N1—C1	1.327 (9)	C6—C7	1.423 (11)
N1—C12	1.359 (9)	C6—H6	0.9500
N2—C10	1.324 (9)	C7—C11	1.404 (10)
N2—C11	1.357 (9)	C7—C8	1.407 (10)
C1—C2	1.392 (12)	C8—C9	1.360 (10)
C1—H1	0.9500	C8—H8	0.9500
C2—C3	1.368 (12)	C9—C10	1.394 (11)
C2—H2	0.9500	C9—H9	0.9500
C3—C4	1.401 (11)	C10—H10	0.9500
C3—H3	0.9500	C11—C12	1.433 (10)
			
N2—Pd1—N1	81.5 (2)	C6—C5—C4	121.1 (7)
N2—Pd1—Cl2	175.11 (17)	C6—C5—H5	119.5
N1—Pd1—Cl2	93.93 (17)	C4—C5—H5	119.5
N2—Pd1—Cl1	93.20 (17)	C5—C6—C7	122.3 (7)
N1—Pd1—Cl1	174.64 (17)	C5—C6—H6	118.8
Cl2—Pd1—Cl1	91.42 (7)	C7—C6—H6	118.8
C1—N1—C12	118.4 (7)	C11—C7—C8	116.1 (7)
C1—N1—Pd1	129.4 (5)	C11—C7—C6	118.5 (7)
C12—N1—Pd1	112.2 (4)	C8—C7—C6	125.4 (7)
C10—N2—C11	118.4 (6)	C9—C8—C7	120.2 (7)
C10—N2—Pd1	129.1 (5)	C9—C8—H8	119.9
C11—N2—Pd1	112.6 (5)	C7—C8—H8	119.9
N1—C1—C2	122.2 (8)	C8—C9—C10	119.8 (7)
N1—C1—H1	118.9	C8—C9—H9	120.1
C2—C1—H1	118.9	C10—C9—H9	120.1
C3—C2—C1	120.0 (7)	N2—C10—C9	122.1 (7)
C3—C2—H2	120.0	N2—C10—H10	119.0
C1—C2—H2	120.0	C9—C10—H10	119.0
C2—C3—C4	119.6 (7)	N2—C11—C7	123.5 (7)
C2—C3—H3	120.2	N2—C11—C12	116.6 (6)
C4—C3—H3	120.2	C7—C11—C12	119.9 (7)
C3—C4—C12	116.8 (7)	N1—C12—C4	122.9 (6)
C3—C4—C5	125.1 (7)	N1—C12—C11	117.0 (6)
C12—C4—C5	118.1 (7)	C4—C12—C11	120.1 (7)
			
N2—Pd1—N1—C1	−177.2 (7)	Pd1—N2—C10—C9	177.3 (5)
Cl2—Pd1—N1—C1	4.4 (7)	C8—C9—C10—N2	1.3 (12)
N2—Pd1—N1—C12	3.4 (5)	C10—N2—C11—C7	0.5 (11)
Cl2—Pd1—N1—C12	−174.9 (5)	Pd1—N2—C11—C7	−178.0 (6)
N1—Pd1—N2—C10	178.1 (7)	C10—N2—C11—C12	−178.3 (6)
Cl1—Pd1—N2—C10	−1.4 (6)	Pd1—N2—C11—C12	3.1 (8)
N1—Pd1—N2—C11	−3.6 (5)	C8—C7—C11—N2	−0.4 (11)
Cl1—Pd1—N2—C11	176.9 (5)	C6—C7—C11—N2	−179.5 (7)
C12—N1—C1—C2	0.8 (11)	C8—C7—C11—C12	178.4 (7)
Pd1—N1—C1—C2	−178.5 (6)	C6—C7—C11—C12	−0.7 (10)
N1—C1—C2—C3	0.1 (13)	C1—N1—C12—C4	−0.9 (10)
C1—C2—C3—C4	−1.0 (12)	Pd1—N1—C12—C4	178.6 (5)
C2—C3—C4—C12	0.9 (11)	C1—N1—C12—C11	177.8 (6)
C2—C3—C4—C5	−179.6 (8)	Pd1—N1—C12—C11	−2.8 (8)
C3—C4—C5—C6	178.3 (7)	C3—C4—C12—N1	0.0 (11)
C12—C4—C5—C6	−2.2 (11)	C5—C4—C12—N1	−179.5 (7)
C4—C5—C6—C7	1.1 (12)	C3—C4—C12—C11	−178.6 (7)
C5—C6—C7—C11	0.3 (11)	C5—C4—C12—C11	1.8 (10)
C5—C6—C7—C8	−178.7 (8)	N2—C11—C12—N1	−0.2 (10)
C11—C7—C8—C9	0.7 (11)	C7—C11—C12—N1	−179.2 (6)
C6—C7—C8—C9	179.7 (7)	N2—C11—C12—C4	178.5 (6)
C7—C8—C9—C10	−1.1 (12)	C7—C11—C12—C4	−0.5 (10)
C11—N2—C10—C9	−1.0 (11)		

### Hydrogen-bond geometry (Å, °)

**Table:**

*D*—H···*A*	*D*—H	H···*A*	*D*···*A*	*D*—H···*A*
C2—H2···Cl1^i^	0.95	2.80	3.733 (11)	169

Symmetry codes: (i) −*x*+1, *y*−1/2, −*z*+1/2.
